# A programme of faculty development in medical education for junior doctors: The start of a journey to clinical educator

**DOI:** 10.12669/pjms.41.5.11936

**Published:** 2025-05

**Authors:** Charles Thurston, Anna Schmid, Chaudhry Aqeel Safdar

**Affiliations:** 1 Dr. Charles Thurston, MBChB, MSc Clinical Teaching Fellow, Clinical and Communication Skill Unit, Institute of Health Sciences Education, Faculty of Medicine and Dentistry, Queen Mary University of London, UK. E1 2AD; 2 Dr. Anna Schmid, MD (Germany) Clinical Lecturer, Clinical and Communication Skill Unit, Institute of Health Sciences Education, Faculty of Medicine and Dentistry, Queen Mary University of London, UK. E1 2AD; 3 Dr. Chaudhry Aqeel Safdar, MBBS, FRCSEd, MSc, MCPS-HPE Senior Clinical Lecturer, Clinical and Communication Skill Unit, Institute of Health Sciences Education, Faculty of Medicine and Dentistry, Queen Mary University of London, UK. E1 2AD

**Keywords:** Early career doctors, Faculty development, Junior doctors

## Abstract

**Objective:**

Interest in medical education for early career doctors is on the rise, but medical education training is insufficient. Medical education faculty development programmes for Junior/Foundation year doctors (JDs) are needed.

**Methods:**

A group of clinical educators designed a development programme and interested Junior doctors were recruited to a pilot. A pre-programme questionnaire established teaching practices and needs of JDs. It had two parts. First included a full day of hands-on workshop, imparting educational principles, teaching skills, developing a lesson plan and practicing structured feedback. The second part invited them to deliver faculty observed skills sessions to medical students, with feedback. Faculty reflection and peer review informed evaluation of the course.

**Results:**

Twelve JDs were recruited, with nine attending (75% retention). Eight completed pre-course questionnaire (89%, n=8/9). This showed frequent bedside teaching (87%, n=7/8), confidence (75%, n=6/8) but ‘not often’ satisfaction with their teaching (75%, n=6/8). JDs’ engagement with post-course evaluation was low (25%). Faculty reflection was a positive approach and generated ideas for improvement.

**Conclusions:**

JDs had high levels of engagement with students, but confidence did not translate into satisfaction, with knowledge and skills gaps. Reflection and peer review provided good insights into the programme and its need. Such programmes are feasible to continue faculty development of JDs, who regularly teach medical students on the wards and can provide clinical educationalist of tomorrow.

## INTRODUCTION

Faculty development is an important part of developing clinical educators. Previously the assumption has been that every doctor is able to teach. As a result, they have been given little support on how to teach.^1^ Early career doctors are an important part of the faculty, more involved in teaching medical students and thus their development remains as important as seniors’ professional development^2^. It has been inconsistent and unstructured.^1^

The role of the clinical teacher is specified in many post-graduate curricula, including the UK’s GMCs *Good Medical Practice* which emphasises the professional duty to teach. After graduation, UK doctors undertake two years generalist training known as the Foundation Programme (FP). The Foundation curriculum has a high-level outcome dedicated to teaching that all doctors must achieve in order to progress.^3^

There has been a rise in the number of junior doctors seeking out educational opportunities^4^ or embark on this new trajectory, altogether.^5^ But there are advantages to development of teaching skills within their early years; taste of medical education, experience of teaching, receiving feedback, and developing a portfolio.^6^

Junior doctors are key stakeholders in undergraduate medical education. They often represent the group of doctors most in contact with the students. This is demonstrated in analogous findings in America where resident doctors contributed more to the cognitive development of medical students than consultants.^1^ Medical schools benefit from high quality teaching on the wards from a quality perspective. Teaching hospitals and Trusts will benefit from promoting the development of their doctors. Ultimately, better teaching and learning for students is a benefit for patients.

The objective of this work was to design an introductory programme in line with principles of faculty development for Junior doctors (JDs).

## METHODS

This programme of ‘Train the Trainers (TtT)’ has been developed and conducted at the Bart’s and the London Medical School of Queen Mary University of London, to support junior/foundation year doctors of Homerton NHS Trust of London, from November 2023 to May 2024.

### Ethical Approval:

Permission was granted by the Institute of Health Sciences Education Internal Ethics and Peer Review committee (reference IPREC280324, dated March 28, 2024).

The authors identified key areas with high yield and most applicable for junior doctors: medical education concepts, clinical teaching, feedback, and designing a lesson plan. We then provided feedback on their teaching sessions. Learning materials were supplied and the programme reviewed by senior medical school faculty.

A pre-course questionnaire was designed to understand JDs current teaching practices and views on teaching and learning. A post-course questionnaire followed to evaluate the programme. A combination of Likert-scale, binary and multiple-choice questions, with associated free text were used.^7^ The questionnaires were evaluated using the QUAID tool.^8^ The programme was also evaluated by reflections from the authors and from peer review by senior medical school faculty.

## RESULTS

A 1.5-day “Train the Trainer” programme was designed. The learning objectives were to:

- Develop an understanding of common concepts in medical education.
- Develop a common language around clinical teaching concepts to draw together practical advice and pedagogy.
- Apply the theoretical knowledge to hands-on teaching on the ward and in medical school.
- Support doctors in forming a community of medical educators, dedicated to providing high quality teaching and educationalist workforce of tomorrow.

The programme was split into a half day seminar session first, followed by a full day of observed clinical skills teaching, with feedback and reflection. A total of 12 JDs were recruited, with nine joining (75%) to attend workshop and then a day of clinical skills teaching, around their clinical commitments.

Eight of these nine doctors completed the pre-course questionnaire, resulting in 89% response rate. Eighty-seven percent (n=7/8) ‘frequently’ or ‘very frequently’ were expected to teach students, and the same number 87% (n=7/8) identified they ‘frequently’ sought out opportunities to teach. Most of the doctors identified ‘bedside teaching’ as their main mode 87% (n=7/8). Six (75%, n=6/8) reported ‘low availability’ of time to teach (occasionally n=3/8, and rarely n=3/8).

The majority of doctors reported ‘not received’ enough training to be an effective teacher (87%, n=7/8), and 87% (n=7/8) responded that they ‘would benefit’ from more formal teacher training. Satisfaction with own teaching was low, with 75% (n=6/8) reporting ‘occasionally’ being satisfied with their teaching. Confidence with teaching was positive; 75% (n=6/8) said they were ‘confident’ about delivering teaching. Confidence with incorporating teaching methods or styles into day-to-day work was more mixed, with only 37% (n=3/8) responding ‘confident’, 25% (n=2/8) ‘neutral’, 37% (n=3/8) ‘unsure’. Fifty percent had low awareness of educational theory (unaware n=3/8, very unaware n=1/8).

There was a spread in frequency of giving structured feedback, 37% (n=3/8) reported giving feedback ‘frequently’, 37% ‘occasionally’ (n=3/8), and 25% (n=2/8) ‘rarely’. There was less awareness of models of feedback, with 50% (n=4/8) ‘unfamiliar’. With regards to session planning, 50% (n=4/8) were ‘comfortable’, 37% (n=3/8) ‘neutral’, and 12% (n=1/9) responding ‘uncomfortable’.

The post-course evaluation questionnaire had a low response rate (n=2/9). Both respondents said they ‘would recommend the course’ to a colleague. Feedback from a senior faculty observer highlighted this was a tutor-led session, at an appropriate level for JDs, and that it could be incorporated into a programme of faculty development targeted at JDs. Suggestions were made to include an element of assessment of learning.

## DISCUSSION

The pre-course questionnaire highlights that JDs have a high level of engagement with students in the clinical environment. There was a good level of confidence while teaching, but this did not necessarily translate into satisfaction with their teaching. This may be the result of a lack of medical education to understand goals and aims to achieve satisfaction with teaching. It may also be related to overall low morale of doctors currently practicing, particularly affected when service provision is prioritised over training opportunities.^9^ Areas that this group of doctors highlighted as weaknesses were medical education theory, evidenced teaching methods and models of feedback.

Feedback and reflection from the authors led to discussions about adapting the course to incorporate the flipped-classroom approach, to facilitate a student-led session and encourage reflection and generation of materials. This could be best facilitated by utilising a virtual learning environment. Assessment of or for learning could be incorporated into such programmes. Overall, self- and peer evaluation was able to identify strengths and weaknesses of the programme and provide a good framework of evaluation independent of other sources of feedback.

As noted in the literature, faculty development programmes are often limited by the time available to the participants^10^, which was demonstrated in this programme with a drop out of 25% of the cohort even before the start of the programme to prioritise service delivery. Further work is needed to develop robust systems between medical schools and education providers to allow time for JDs to continue their development.

This programme analysis is limited by small cohort sizes, which particularly affects the post-course evaluation. Engagement may have been low because of their time constraint and low priority given to evaluation in a busy clinical rota. Further methods to engage JDs in evaluation are needed; like releasing certificates of attendance after the completion of the post-evaluation survey.

## CONCLUSION

Faculty development of clinical teachers is an important part of medical education, and JDs remain a core part of the extended faculty. Programmes like ours are replicable and feasible across undergraduate and postgraduate medical education to start the journey of early career doctors becoming clinical educators and join the much-needed future educationalists workforce.
